# The role of gibberellins in improving the resistance of tebuconazole-coated maize seeds to chilling stress by microencapsulation

**DOI:** 10.1038/srep35447

**Published:** 2016-11-07

**Authors:** Lijuan Yang, Daibin Yang, Xiaojing Yan, Li Cui, Zhenying Wang, Huizhu Yuan

**Affiliations:** 1 Key Laboratory of Integrated Pest Management in Crops, Ministry of Agriculture, Institute of Plant Protection, Chinese Academy of Agricultural Sciences, Beijing, 100193, China

## Abstract

Chilling stress during germination often causes severe injury. In the present study, maize seed germination and shoot growth under chilling stress were negatively correlated with the dose of tebuconazole in an exponential manner as predicted by the model Y = A + B × e^(−x/k)^. Microencapsulation was an effective means of eliminating potential phytotoxic risk. The gibberellins (GAs) contents were higher after microencapsulation treatment than after conventional treatment when the dose of tebuconazole was higher than 0.12 g AI (active ingredient) kg^−1^ seed. Further analysis indicated that microencapsulation can stimulate ent-kaurene oxidase (KO) activity to some extent, whereas GA 3-oxidase (GA3ox) and GA 2-oxidase (GA2ox) activities remained similar to those in the control. Genes encoding GA metabolic enzymes exhibited different expression patterns. Transcript levels of *ZmKO1* increased in the microcapsule treatments compared to the control. Even when incorporated into microcapsules, tebuconazole led to the upregulation of *ZmGA3ox1* at doses of less than 0.12 g AI kg^−1^ seed and to the upregulation of *ZmGA3ox2* when the dose was higher than 0.12 g AI kg^−1^ seed. With increasing doses of microencapsulated tebuconazole, the transcript levels of *ZmGA2ox4*, *ZmGA2ox5* and *ZmGA2ox6* exhibited upward trends, whereas the transcript levels of *ZmGA2ox7* exhibited a downward trend.

Maize (*Zea mays* L.) is a globally important crop. Maize is used widely not only for human food but also as a basic ingredient in animal feed and as a raw material for the manufacturing of many industrial products^1^. Maize is susceptible to chilling stress and requires warm temperatures for seed germination and shoot growth^2^. The susceptibility of maize to chilling stress varies among varieties. Maize seeds may not germinate at temperatures below 10–17 °C^2^,^3^,^4^.Global warming and breeding efforts to improve the chilling tolerance of maize have extended maize cultivation northwards. In northern areas, the maize temperature requirement is not always fulfilled^5^. Suboptimal temperatures during germination in the spring often cause severe chilling injury in maize^6^. Moreover, with the trend towards more frequent and extreme weather events, sudden and unexpected chilling stress after seed planting often has negative impacts on maize seed germination and shoot growth^7^.

Chilling stress has been reported to suppress seed germination and shoot growth, delay the onset and cessation of emergence, and extend the duration of emergence^8^. Seed treatment is a common agricultural practice to protect crops from attack by pest insects and diseases. If active ingredients employed for seed treatment have plant growth-retarding effects, phytotoxicity caused by seed treatment might be worse under chilling stress.

Tebuconazole is a triazole fungicide that is widely applied as a seed treatment for protecting maize from head smut (*Sphacelotheca reiliana*). Tebuconazole also possesses plant growth-regulating properties^9^. At inappropriate doses, tebuconazole can reduce seed germination and inhibit plant growth^10^,^11^. Even at recommended doses, the use of triazole fungicides as a seed treatment can threaten the normal growth of maize shoots under chilling stress^10^. For example, in northern China, phytotoxicity caused by seed-coating with triazole fungicides occurs occasionally in maize under low-temperature conditions after planting, with an incidence of 10% to 30% in 2008^12^. The negative effects of triazole fungicides appear to be related to gibberellin (GA) biosynthesis^9^,^13^,^14^. However, triazole compounds can also promote plant growth under some conditions. For example, Gopi, *et al.*^15^ observed that hexaconazole and paclobutrazol increase both the fresh weight and dry weight of carrot plants.

Growth retardation is one adaptation of plants to chilling stress^16^. GAs regulate seed dormancy, plant growth and development^17^. The GA signalling pathway modulates plant growth and plays an important role in adaptation to stress conditions^18^,^19^,^20^. Under chilling stress, deactivation of GAs is enhanced, levels of bioactive GAs are decreased, and levels of inactive hydroxylated forms are elevated, leading to rapid growth suppression^21^. However, normal growth can be resumed after exogenous GA_3_ treatment under chilling stress^22^. Therefore, exogenous GAs have been used in an attempt to reduce the risk of negative effects of triazole^11^,^23^,^24^. However, exogenous GAs can significantly stimulate shoot growth and may cause plant lodging at late stages^25^,^26^. Consequently, the combination of exogenous GAs treatments and seed treatment is not widely practiced.

The suppressive effects of tebuconazole on plants appear to be dose dependent^11^,^27^. Yang, *et al.*^28^ observed that microencapsulation of seed-coating tebuconazole was superior to conventional formulations because of its advantages in enhancing shoot emergence, stimulating maize shoot growth, increasing photosynthetic pigment contents, and improving the bioefficacy of controlling maize head smut at normal growth temperature. These positive effects of microencapsulated tebuconazole are associated with changes in the phytohormone balance between GAs and abscisic acid (ABA). However, whether microencapsulation can improve the tolerance of tebuconazole-coated maize seeds to chilling stress is unknown.

This study aimed to study the effect of microencapsulated tebuconazole on the resistance of maize seeds to chilling stress. We investigated the effects of coating maize seeds with microencapsulated tebuconazole on maize seed germination and the responses of GA metabolic enzymes and regulatory genes.

## Results

### Germination rate of maize seeds

The germination rate is a key factor used to evaluate the safety of chemicals used as seed coatings. As shown in Table 1, the germination rate of conventional treatments after chilling stress gradually decreased with increasing doses of tebuconazole. At a dose of 0.6 g AI kg^−1^ seed, the germination rate was only 64.4%. Statistically, the seed germination rate was negatively correlated with the dose of tebuconazole, as described by the following exponential model:


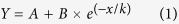


where *Y* is the germination rate, *X* is the dose of tebuconazole, and *A*, *B*, and *k* are constants. The calculated values of *A*, *B*, and *k* were 98.25, −1.14 and −0.18, respectively, with *r* = 0.9878.

However, microencapsulation of tebuconazole eliminated this suppressive effect under chilling stress. The germination rate of the microencapsulated formulation treatment was greater than 95.6% at the tested doses and was also higher than that of conventional tebuconazole treatments at the same dose.

### Maize growth

**Figure 1:**
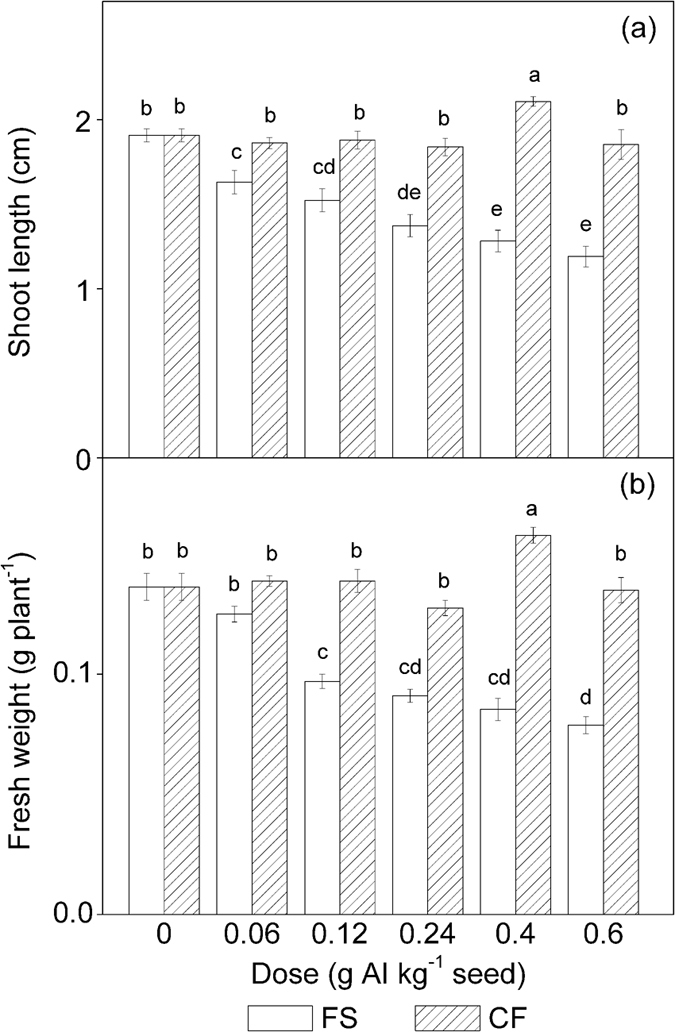
The length and fresh weight of maize shoots grown from seeds coated with different doses of tebuconazole under chilling stress. AI = active ingredient, FS = flowable concentrate for seed treatment, CF = capsule suspension for seed treatment. The results were presented as the means ± SE. The control treatment was set at zero. Different letters above the bars indicated significant differences at *P* < 0.05 (one-way ANOVA, Duncan’s multiple range test).

As shown in Fig. 1, the length and fresh weight of shoots developed from tebuconazole-coated seeds were gradually suppressed with an increasing dose of conventional tebuconazole. At a dose of 0.6 g AI kg^−1^ seed, the shoot length and fresh weight of the shoots were reduced by 37.6% and 42.2% compared to the untreated control plants, respectively. Regression analysis revealed that this dose-dependent suppression also satisfied an exponential model (1). For shoot length, the calculated values of *A*, *B*, and *k* were 1.21, 0.68 and 0.16, respectively, with *r* = 0.9906. For shoot fresh weight, the calculated values of *A*, *B*, and *k* were 0.080, 0.059 and 0.14, respectively, with *r* = 0.9614.

However, negative effects on maize shoots caused by conventional tebuconazole under chilling stress were not observed in the microencapsulated tebuconazole treatments. The shoot length and fresh weight in the microcapsule treatments were not significantly different from those of the control (*P* > 0.05). At a dose of 0.4 g AI kg^−1^ seed, the shoot length and fresh weight of the microcapsule treatments were significantly higher than those of the control shoots (*P* < 0.05) (Fig. 1).

### Determination of GA content

**Figure 2:**
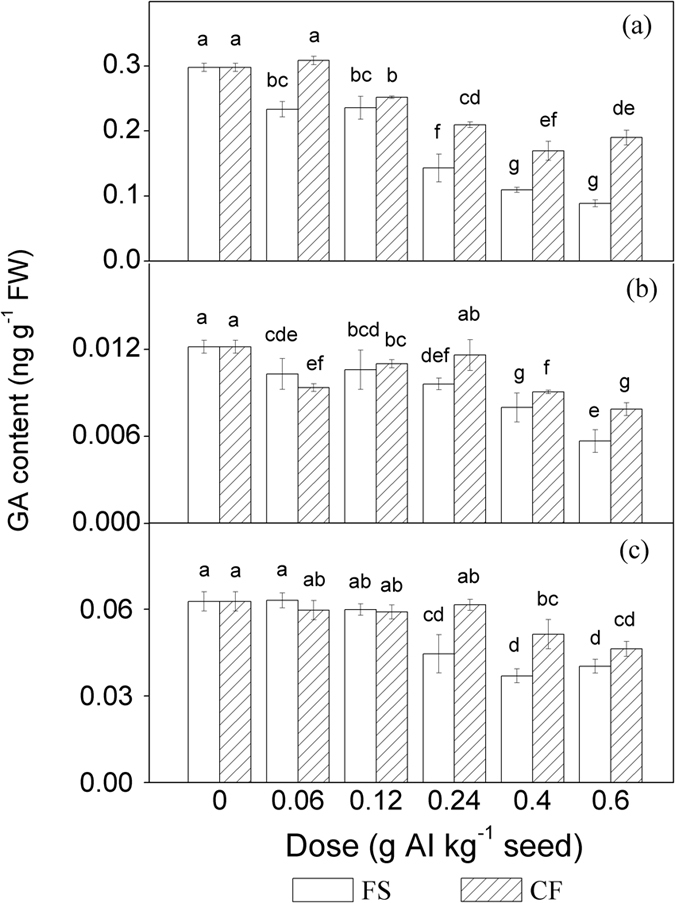
GA content of maize shoots grown from seeds coated with different doses of tebuconazole under chilling stress. (**a**) GA_3_ content. (**b**) GA_4_ content, (**c**) GA_1_ content. AI = active ingredient, FW = fresh weight, FS = flowable concentrate for seed treatment, CF = capsule suspension for seed treatment. The results were presented as the means ± SE. The control treatment was set at zero. Different letters above the bars indicated significant differences at *P* < 0.05 (one-way ANOVA, Duncan’s multiple range test).

As shown in Fig. 2, the GA_1_, GA_3_ and GA_4_ content exhibited downward trends as the doses of conventional tebuconazole increased. The GA_3_ content of the conventional treatments was significantly lower than that of the control when the dose of tebuconazole was higher than 0.06 g AI kg^−1^ seed (*P* < 0.05). At a dose of 0.6 g AI kg^−1^ seed, the GA_3_ content of the conventional treatments was decreased by 70.2% (Fig. 2a).The GA_1_ and the GA_4_ contents of the conventional treatments were lower than those of the control, but the differences were not statistically significant when the dose of tebuconazole was less than 0.12 g AI kg^−1^ seed (*P* > 0.05). At a dose of 0.6 g AI kg^−1^ seed, the GA_1_ and the GA_4_ contents of the conventional treatments were reduced by 52.5% and 36.0% relative to the control, respectively (Fig. 2b,c).

However, in the tested dose range (0.06–0.6 g AI kg^−1^ seed), the GA_3_ content of the microcapsule treatments was higher than that of the conventional treatments at the same dose of tebuconazole. When the doses of tebuconazole were higher than 0.12 g AI kg^−1^ seed, the GA_1_ contents of the microcapsule treatments were higher than those of the conventionally treated maize at the same dose of tebuconazole. When the doses of tebuconazole were higher than 0.24 g AI kg^−1^ seed, the GA_4_ contents of the microcapsule treatments were higher than those of the conventionally treated maize at the same dose of tebuconazole.

### Expression analysis of GA metabolic enzyme genes

**Figure 3:**
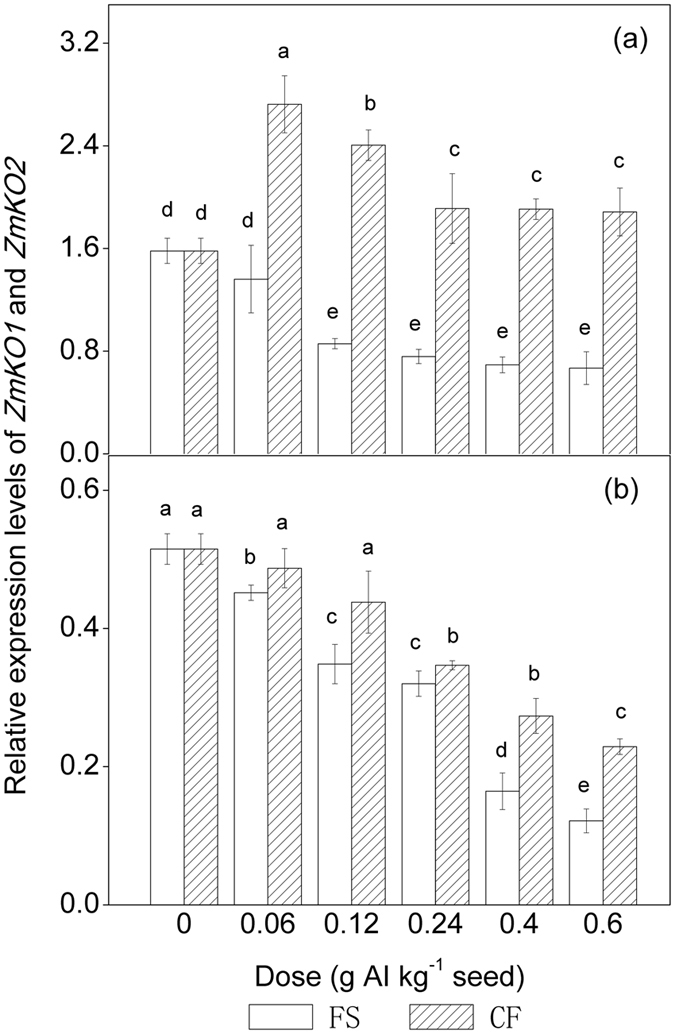
Relative mRNA expression levels of *ZmKO1* and *ZmKO2* encoding KO oxidase in maize shoots coated with tebuconazole under chilling stress. (**a**) Relative mRNA expression levels of *ZmKO1*. (**b**) Relative mRNA expression levels of *ZmKO2*. AI = active ingredient, FS = flowable concentrate for seed treatment, CF = capsule suspension for seed treatment. The results were presented as the means ± SE. The control treatment was set at zero. Different letters above the bars indicated significant differences at *P* < 0.05 (one-way ANOVA, Duncan’s multiple range test).

KO is a multifunctional cytochrome P450 enzyme that catalyses three-step oxidation of ent-kaurene to ent-kaurenoic acid in the GA biosynthetic pathway. The KO in maize is encoded by *ZmKO1* and *ZmKO2*^29^. The relative expression levels of *ZmKO1* and *ZmKO2* exhibited a downward trend with increasing conventional tebuconazole doses (Fig. 3). Regression analyses further indicated that the relative expression levels of *ZmKO1* and *ZmKO2* were negatively correlated with the dose of conventional tebuconazole. An exponential model (1) can also be applied to describe this dose-dependent suppression (*ZmKO1*: *r* = 0.9901, *ZmKO2*: *r* = 0.9810). Similarly, the relative expression levels of *ZmKO1* and *ZmKO2* also exhibited an exponential downward trend with increasing microencapsulated tebuconazole dose (*ZmKO1*: *r* = 0.9734, *ZmKO2*: *r* = 0.9945). However, the relative expression levels were higher in the microcapsule treatments than the conventional treatments at the same dose of tebuconazole. In particular, the mRNA levels of *ZmKO1* in the microcapsule treatments were higher than those in the untreated control (Fig. 3a). This result demonstrated that a small amount of free tebuconazole can stimulate the expression of *ZmKO1* to some extent in germinated maize seeds. However, the mRNA levels of *ZmKO2* in the microcapsule treatments were not significantly different from those in the untreated control at a dose of 0.06–0.12 g AI kg^−1^ seed (*P* > 0.05) (Fig. 3b).

**Figure 4:**
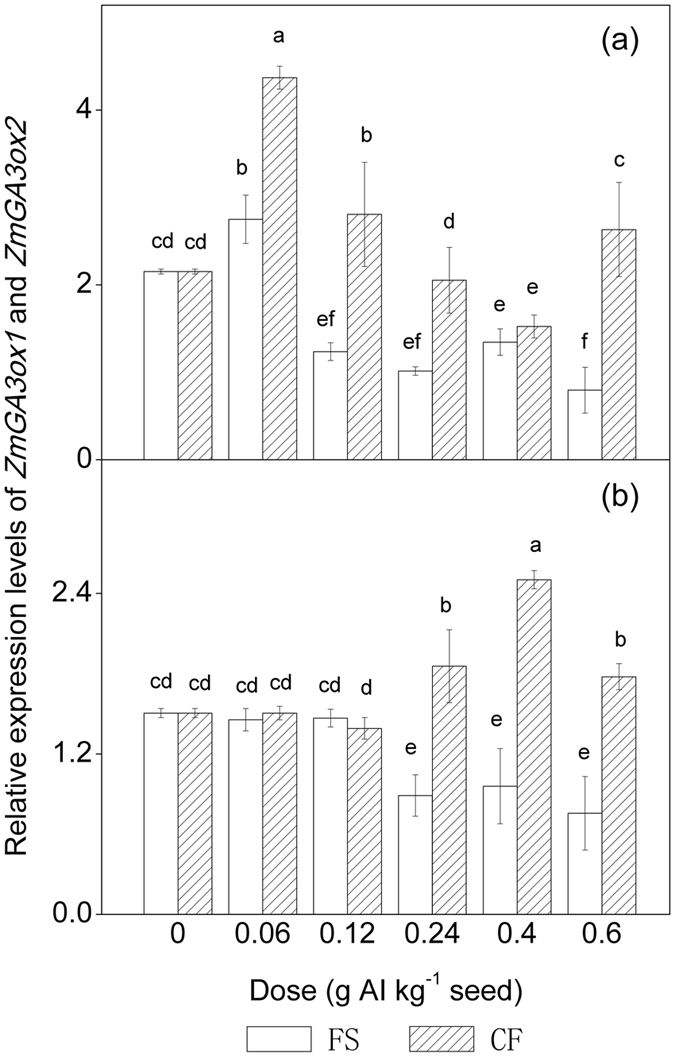
Relative mRNA expression levels of *ZmGA3ox1* and *ZmGA3ox2* encoding GA 3-oxidase in maize shoots coated with tebuconazole under chilling stress. (**a**) Relative mRNA expression levels of *ZmGA3ox1*. (**b**) Relative mRNA expression levels of *ZmGA3ox2*. AI = active ingredient, FS = flowable concentrate for seed treatment, CF = capsule suspension for seed treatment. The results were presented as the means ± SE. The control treatment was set at zero. Different letters above the bars indicated significant differences at *P* < 0.05 (one-way ANOVA, Duncan’s multiple range test).

The genes encoding GA3ox in GA biosynthesis, *ZmGA3ox1* and *ZmGA3ox2*, have been identified in maize^29^. As shown in Fig. 4, the relative expression levels of *ZmGA3ox1* and *ZmGA3ox2* exhibited a dose-dependent suppression trend in the conventional tebuconazole treatments. Further study revealed that conventional tebuconazole increased the expression of *ZmGA3ox1* at 0.06 g AI kg^−1^ seed. However, compared with the control, microencapsulated tebuconazole stimulated the expression of *ZmGA3ox1* and *ZmGA3ox2* in newly developed shoots. Microencapsulated tebuconazole stimulated the expression of *ZmGA3ox1* at lower doses. By contrast, microencapsulated tebuconazole stimulated the relative expression of *ZmGA3ox2* when the tested dose was higher than 0.12 g AI kg^−1^ seed.

**Figure 5:**
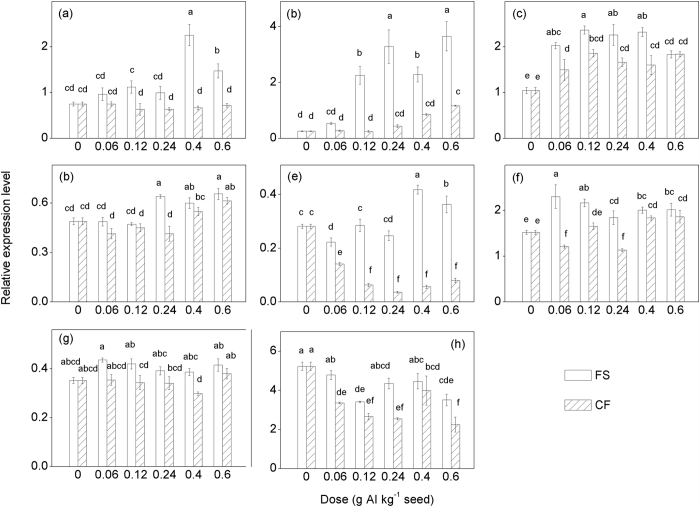
Relative mRNA expression levels of the genes encoding GA 2-oxidase in maize shoots coated with tebuconazole under chilling stress. (**a**–**h**) Relative mRNA expression levels of *ZmGA2ox1*, and *ZmGA2ox4- ZmGA2ox10*. AI = active ingredient, FS = flowable concentrate for seed treatment, CF = capsule suspension for seed treatment. The results were presented as the means ± SE. The control treatment was set at zero. Different letters above the bars indicated significant differences at *P* < 0.05 (one-way ANOVA, Duncan’s multiple range test).

Ten GA catabolic genes, *ZmGA2ox1-ZmGA2ox10*, encode GA2ox in maize^29^. Transcripts of 8 of the 10 *ZmGA2ox* genes were detectable in new shoots (*ZmGA2ox1*, *ZmGA2ox4*, *ZmGA2ox5*, *ZmGA2ox6*, *ZmGA2ox7*, *ZmGA2ox8*, *ZmGA2ox9* and *ZmGA2ox10*) (Fig. 5). The relative expression levels of these eight *ZmGA2ox* genes revealed different patterns. At the same tested dose, the relative *ZmGA2ox* expression levels were lower in the microencapsulated tebuconazole treatments than in the conventional tebuconazole treatments. For example, the relative expression levels of *ZmGA2ox1*, *ZmGA2ox4* and *ZmGA2ox7* in the microcapsule treatments were only 48%, 32% and 20% of the levels in the conventional treatments at a dose of 0.6 g AI kg^−1^ seed, respectively (Fig. 5a,b,e). Furthermore, four genes (*ZmGA2ox1*, *ZmGA2ox4*, *ZmGA2ox5* and *ZmGA2ox6*) exhibited an upward trend with increasing dose of tebuconazole in the conventional treatments (Fig. 5a–d). Among these four genes, *ZmGA2ox4*, *ZmGA2ox5* and *ZmGA2ox6* also exhibited an upward trend with increasing dose of microencapsulated tebuconazole. Interestingly, the relative expression levels of *ZmGA2ox7* exhibited an upward trend in the conventional tebuconazole treatments but a downward trend in the microencapsulated tebuconazole treatments (Fig. 5e).

### GA catabolic enzyme activity analysis

**Figure 6:**
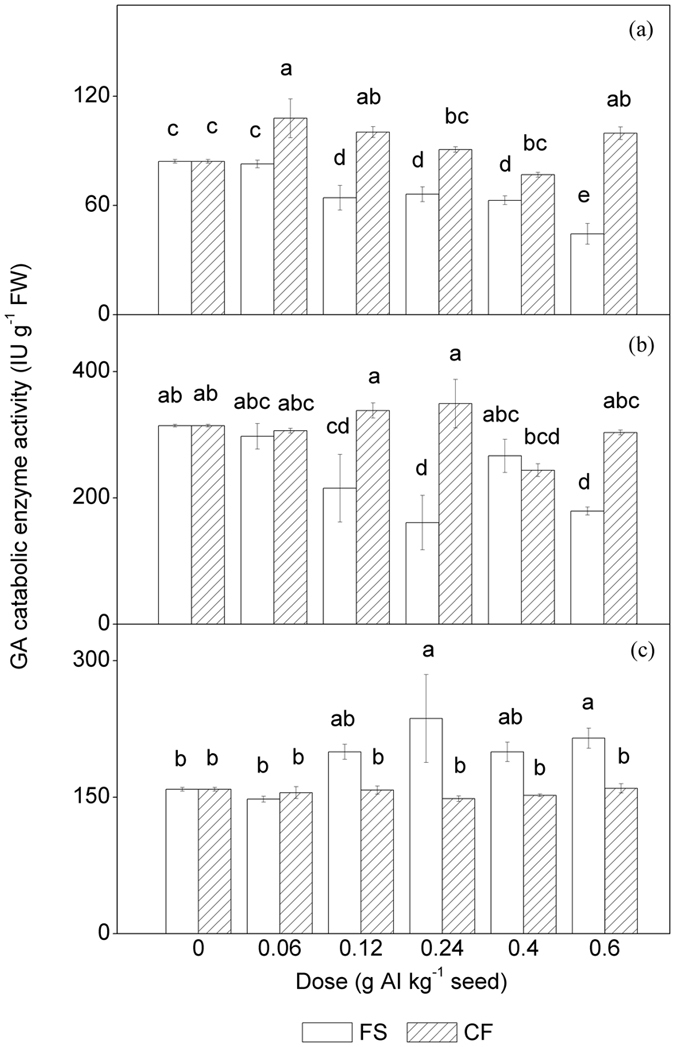
Enzyme activities of the maize shoots coated with different doses of tebuconazole under chilling stress. (**a**) KO activity, (**b**) GA3-oxidase activity, (**c**) GA3-oxidase activity. AI = active ingredient, FW = fresh weight, FS = flowable concentrate for seed treatment, CF = capsule suspension for seed treatment. The results were presented as the means ± SE. The control treatment was set at zero. Different letters above the bars indicated significant differences at *P* < 0.05 (one-way ANOVA, Duncan’s multiple range test).

As shown in Fig. 6a, the KO activity in the conventional treatments was significantly suppressed when the dose was greater than 0.06 g AI kg^−1^ seed. At a dose of 0.6 g AI kg^−1^ seed, the KO activity of the conventional treatment was only 52.68% of that in the control. However, the microencapsulated tebuconazole can stimulate the KO activity to some extent. The microencapsulated tebuconazole increased the KO activity by 28.1% at a dose of 0.06 g AI kg^−1^ seed and 18.3% at a dose of 0.6 g AI kg^−1^ seed compared to the control.

The GA3ox activity of maize shoots was suppressed by conventional tebuconazole in a similar manner as the KO activity (Fig. 6b). At a dose of 0.6 g AI kg^−1^ seed, GA3ox activity was reduced by 43.03% compared to that of the control. However, the suppression caused by conventional tebuconazole was eliminated by microencapsulation. The GA3ox activities in shoots grown from microencapsulated tebuconazole-treated seeds were similar to those in the control.

By contrast, conventional tebuconazole stimulated the activity of GA2ox (Fig. 6c). At doses of 0.24 and 0.6 g AI kg^−1^ seed, GA2ox activities were significantly increased by 49% and 35.37%, respectively (*P* < 0.05), but the GA2ox activities of the microencapsulated tebuconazole treatments were not significantly different from those of the control (*P* > 0.05).

## Discussion

Triazole fungicides inhibit the biosynthesis of gibberellin (GA), thus altering the phytohormone balance in plant tissues and inhibiting seed germination and plant growth^9^,^13^,^14^. Chilling temperatures during seed germination increase the risk of phytotoxicity caused by seed-coating triazole treatments. The stress temperature in our work was close to the low temperature limit that maize may encounter in spring. A previous study by Wang, *et al.*^10^ indicated that this low temperature can significantly suppress maize growth when maize seeds are coated with triazole compounds. In our study, multivariate analysis of variance (MANOVA) of the whole data sets (Table 2) indicated that the maize seed germination rate and shoot growth were significantly affected by both the dose and formulation as well as their interaction (*P* < 0.05). Under chilling stress, the maize seed germination rate, shoot length and fresh weight were negatively correlated with the dose in the tested dose range (0.06–0.6 g AI kg^−1^ seed) when their correlations were analysed by applying exponential model (1) (Fig. 1). The recommended dose of tebuconazole is 0.06–0.12 g AI kg^−1^ seed. This result indicates that tebuconazole should not be overdosed for seed treatment and that uniformly coating the seed is essential. In a study by Yang, *et al.*^28^, microencapsulated tebuconazole stimulated maize seed germination and shoot growth to some extent at doses of 0.04–1.0 g AI kg^−1^ seed. However, the effects of microencapsulated tebuconazole on maize seed germination and shoot growth under chilling stress have remained unclear. In our study, the germination rate, shoot length and fresh weight after microencapsulated tebuconazole treatment were not significantly different from those of the control plants in the tested dose range but were higher than those in the conventional treatments at the same dose of tebuconazole (Fig. 1, Table 1). This result suggests that microencapsulation of tebuconazole is an effective way to overcome the detrimental effects of tebuconazole. The beneficial effects of microencapsulation are attributable to the reduction of the dose of free tebuconazole that seeds and plants contact directly, and this sustained exposure to a low dose of tebuconazole does not have adverse effects on maize seed germination and shoot growth.

GA plays a vital role in regulating seed dormancy, plant growth and development^17^. Triazole compounds can inhibit GA biosynthesis^30^. Moreover, chilling stress can also lead to decreased levels of GAs in plant tissues^21^. GA_1_, GA_3_ and GA_4_ are three bioactive GAs. In our study, MANOVA of the data sets (Table 2) indicated that the contents of bioactive GAs were significantly affected by both dose and formulation as well as their interaction (*P* < 0.05). Under the chemical stress of tebuconazole and chilling stress, the GA_1_, GA_3_ and GA_4_ contents were lower in the maize shoots in the conventional treatments than in the control, with a downward trend with increasing dose of tebuconazole (Fig. 2). However, microencapsulation restored bioactive GAs levels in maize shoots to some extent compared to conventional treatments under chilling stress. These results demonstrate that microencapsulation can effectively alleviate the risk of phytotoxicity when coating with tebuconazole.

**Figure 7:**
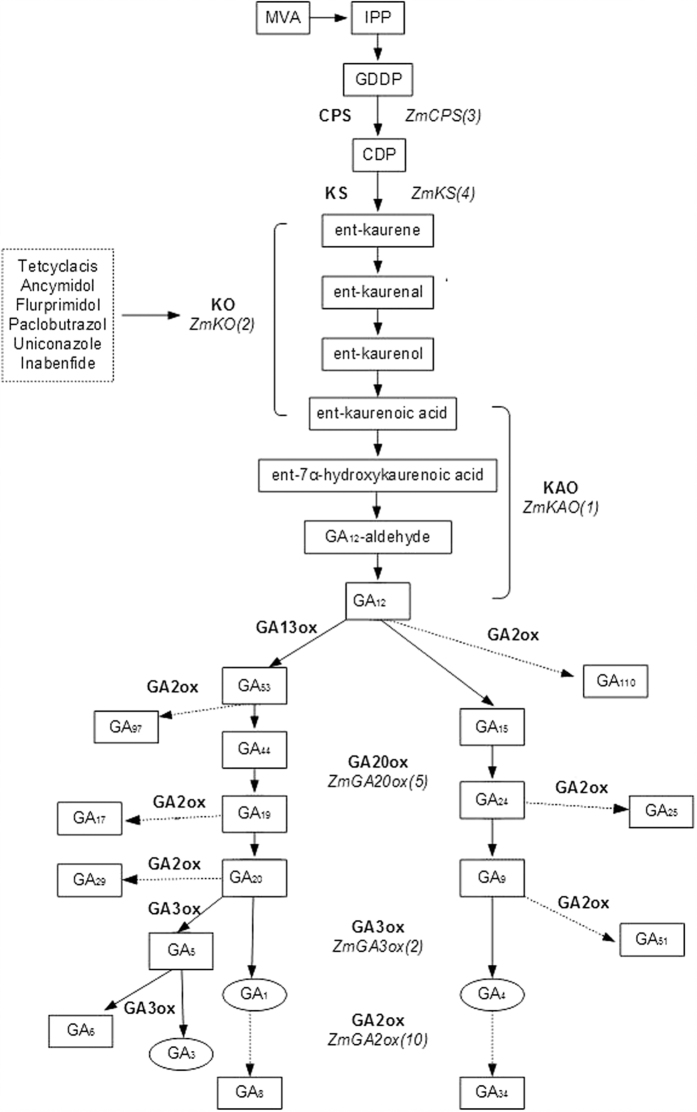
GA biosynthesis in maize. Solid arrows: metabolic steps in GA biosynthesis; dashed-line arrows: inactivation steps; bold words: enzymes catalysing these reactions; italics: maize gene names and their numbers; dashed-box structure: inhibition of the indicated enzyme; circled structures: bioactive GAs; brackets: multiple steps catalysed by one enzyme. The oxidization of GA_12_ to GA_53_ is catalysed by GA 13-oxidase, which has not been cloned^43^.

GAs form a large family of tetracyclic diterpenoid phytohormones, and biosynthesis of GA in plants can be divided into seven steps, which are regulated by seven GA metabolic enzymes: ent-copalyl diphosphate synthase (CPS), ent-kaurene synthase (KS), KO, ent-kaurenoic acid oxidase (KAO), GA 20-oxidase (GA20ox), GA3ox, and GA2ox^31^,^32^,^33^,^34^. In maize, the seven GA metabolic enzymes are encoded by 27 genes^29^, and the detailed GA biosynthesis pathway is shown in Fig. 7 29,31,32,35. The KO gene has been reported to be responsible for plant height. For example, a deficiency of KO activity causes a GA-deficient rice mutant (d35^Tan-Ginbozu^)^36^. Loss-of-function mutation in the Arabidopsis KO gene (*ga3*) or pea gene (*lh*) results in dwarf and male-fertile phenotypes^37^,^38^,^39^. Triazoles are inhibitors of GA biosynthesis^14^,^40^,^41^. These compounds are competitive inhibitors of KO^42^. For example, high levels of ent-kaurene are observed in paclobutrazol-treated Arabidopsis^43^. Song, *et al.*^29^ determined that transcript levels of *ZmKO1* and *ZmKO2* were inhibited by paclobutrazol during maize seed germination^29^. In this study, MANOVA of the whole data sets (Table 2) indicated that transcript levels of *ZmKO1* and *ZmKO2 and* the activity of KO were significantly affected by both dose and formulation as well as their interaction (*P* < 0.05). We observed that the expression levels of *ZmKO1* and *ZmKO2* were negatively correlated with the dose of conventional tebuconazole under chilling temperature stress in an exponential manner as predicted by equation (1) (Fig. 3). KO-overexpressing lines of Arabidopsis are more sensitive to paclobutrazol and uniconazole than wild type^41^. In the microencapsulated tebuconazole treatments, although the relative expression levels of *ZmKO1* and *ZmKO2* exhibited an exponential downward trend with increasing microencapsulated tebuconazole dose, the expression levels of *ZmKO1* in the microcapsule treatments were all greater than the levels in untreated plants, and the expression levels of *ZmKO2* were not significantly different from the untreated treatment at a dose of 0.06–0.12 g AI kg^−1^ seed. This result indicates that limited direct exposure of maize seeds to free tebuconazole released from microcapsules can benefit the biosynthesis of KO to some extent.

Although more than 100 GAs have been identified^44^, only a small number, including GA_1_, GA_3_ and GA_4_, are bioactive plant growth regulators^45^. Bioactive GAs are tightly regulated by two metabolic enzymes, GA3ox and GA2ox (Fig. 7). GA3ox catalyses the final steps in the conversion of GA intermediates (GA_5_, GA_20_ and GA_9_) to bioactive GAs (GA_1_, GA_3_ and GA_4_). Teng, *et al.*^26^ revealed that *ZmGA3ox1* and *ZmGA3ox2* function to control the elongation of the vegetative shoot and possibly regulate the production of GA_1_ in maize. A loss-of-function mutation of *ZmGA3ox2 (d1*) exhibits a dwarf phenotype because the metabolism of GA_20_ to GA_1_ is blocked, and further analysis revealed that the GA content in *d1* was less than 2% of that in normal shoots, whereas GA_20_ and GA_29_ accumulated by more than 10-fold compared to normal shoots^46^. In our study, MANOVA of the data sets (Table 2) indicated that the transcript levels of *ZmGA3ox1* and *ZmGA3ox2* as well as the activity of GA3ox were significantly affected by both dose and formulation as well as their interaction (*P* < 0.05). The relative expression levels of *ZmGA3ox1* and *ZmGA3ox2* exhibited a dose-dependent suppression trend in the conventional tebuconazole treatments (Fig. 4). However, the expression patterns of these genes in response to microencapsulated tebuconazole differed. Microencapsulated tebuconazole can stimulate the relative expression of *ZmGA3ox1* only at lower doses. Conventional tebuconazole can also increase the expression of *ZmGA3ox1* at 0.06 g AI kg^−1^ seed. These results indicate that *ZmGA3ox1* is sensitive to tebuconazole exposure. By contrast, the microcapsule treatments did not alter the relative expression of *ZmGA3ox2* at low doses but stimulated its expression when the dose was higher than 0.12 g AI kg^−1^ seed. This observation explains why controlled release of tebuconazole from microcapsules led to higher GA levels than conventional treatments when the dose was higher than 0.12 g AI kg^−1^ seed.

GA2ox plays an important role in plant height. GA2ox converts active GAs and precursors into inactive forms^47^. Silencing GA2ox can increase tobacco growth and fibre production^48^. Loss-of-function mutation in the pea GA2ox gene (*PsGA2ox1*) results in a hyperelongated slender phenotype^49^. Overexpression of the rice GA2ox genes causes a dwarf phenotype and a delay in reproductive development^50^. In maize, the expression patterns of these *ZmGA2ox* genes vary considerably after treatment with paclobutrazol. The transcript levels of *ZmGA2ox1*, *ZmGA2ox3* and *ZmGA2ox10* are upregulated by paclobutrazol^29^. In our study, these ten genes also exhibited complicated expression patterns after exposure to conventional or microencapsulated tebuconazole (Fig. 5). MANOVA of the data sets (Table 2) indicated that most of the transcript levels of *ZmGA2ox* genes were significantly affected by both dose and formulation as well as their interaction (*P* < 0.05). The transcript levels of *ZmGA2ox9* and the activity of GA2ox were not significantly affected by the dose of tebuconazole (*P* > 0.05) but were significantly affected by the formulation (*P* < 0.05). The relative expression levels of *ZmGA2ox5*, *ZmGA2ox9* and *ZmGA2ox10* were not significantly affected by the interaction between doses and formulations (*P* > 0.05). In general, the relative expression levels of GA2ox genes were lower in the microencapsulated tebuconazole treatments than in conventional tebuconazole treatments compared at same tested dose. In particular, the expression levels of *ZmGA2ox7* and *ZmGA2ox10* were significantly downregulated by microencapsulated tebuconazole treatment. By contrast, *ZmGA2ox4*, *ZmGA2ox5* and *ZmGA2ox6* exhibited an upward trend with increasing dose of microencapsulated tebuconazole. Taken together, these results suggest that the biological activity of GA2ox probably remained at the same level in germinated maize seeds treated with microencapsulated tebuconazole as in the untreated control.

In summary, microencapsulation can eliminate the suppressive effect of tebuconazole on maize seeds and shoots under chilling stress. After microcapsule treatment, the GAs contents were higher than those of conventional treatments at a relatively high dose of tebuconazole. The recovery of the GA content was probably due to the combined effects of higher KO and GA3ox activities, which convert GA intermediates into bioactive GAs, and reduced GA2ox activity, which converts active GAs and precursors into inactive forms in maize shoots.

## Methods

### Plant material and growth conditions

Seeds of maize (nonghua 101) were generously supplied by the Da Bei Nong Group (Beijing, China). The conventional flowable concentrate for seed treatment (FS) of tebuconazole (60 g L^−1^) was a gift from Bayer Crop Science AG. The capsule suspension for seed treatment (CF) (encapsulation efficiency >90%) was prepared after the method of Yang, *et al.*^28^.

Seeds were treated with either 60 g L^−1^ FS or CF. Both formulations were applied at rates of 0.06, 0.12, 0.24, 0.4, and 0.6 g AI kg^−1^ seed (AI = active ingredient). All treatments were applied by stirring 100 g of seeds with formulations in 1 mL of water. The seeds in the untreated control were stirred with 1 mL of water.

The coated maize seeds were planted in a mixture of vermiculite and peat moss (1:1) in a greenhouse at 25 °C/20 °C(12 h/12 h, light/dark). At 60 h after planting, the seeds were exposed to chilling in a growth chamber at 17 °C/6 °C (12 h/12 h, light/dark) for 6 d^10^. After the chilling treatments, the germination rate was recorded, and shoots in each treatment were sampled randomly. Shoot length and fresh weight were measured after sampling. Shoots for mRNA expression analysis and GA content determination were frozen in liquid nitrogen and stored at −80 °C.

### Analysis of GA content

The GAs were extracted and purified by the method of Yang, *et al.*^28^ A 5-g mass of fresh samples was extracted and homogenized in 5 mL of methanol:water (80:20). The extract was incubated at 4 °C for 48 h and then centrifuged at 4000 rpm for 15 min at 4 °C. The supernatant was passed through C18 Sep-Pak cartridges (Waters Corp., Milford, MA, USA), and the phytohormone fraction was eluted with 10 mL of methanol and 10 mL of ether. The eluate was dried under pure N_2_ at 20 °C and then resuspended in 100 μL of 100% methanol. 15 μL of each sample was injected into a UPLC/ESI-MS/MS system (Waters, USA), and the eluted ions were monitored by MRM. The GAs contents were assayed using the method of Urbanova, *et al.*^51^.

### Analysis of gene expression by quantitative RT-PCR

Total RNA was extracted using the SV Total RNA Isolation System (Promega Corporation, Madison, WI, USA) according to the manufacturer’s instructions. The RNA quality was assessed by electrophoresis on a 1% agarose gel stained with ethidium bromide. The RNA concentration was measured on a NanoDrop ND2000 spectrophotometer (NanoDrop Technologies). cDNA was synthesized from RNA samples using the PrimeScript^TM^ RT reagent Kit with gDNA Eraser (Perfect Real Time) (Takara, Dalian, China). The gene-specific primers used for quantitative real-time PCR were described in Supporting Information Table S1. After reverse transcription, the cDNA was used as the template for quantitative real-time PCR in an ABI 7500 Real-time PCR System (Applied Biosystems) with SYBR^®^ Premix Ex Taq TM || (Tli RNaseH Plus) (Takara, Dalian, China). The reactions were conducted in 20 μL containing 2 μL of cDNA (100 ng μL^−1^), 10 μL of SYBR Premix Ex Taq™, 0.4 μL of forward primer (10 μM), 0.4 μL of reverse primer (10 μM), 0.4 μL of Rox Reference Dye II(50×) and 6.8 μL of ddH_2_O. The standard PCR conditions for the ABI7500 were used: 95 °C for 30 s and 40 cycles of 95 °C for 5 s and 60 °C for 34 s. After the cycling protocol, melting curve analysis from 60 °C to 95 °C was applied to all reactions to verify the formation of a single PCR product. The quantification results were expressed in terms of the cycle threshold (CT) value determined according to the manually adjusted baseline. The amplification efficiency of genes was estimated using E = (10^−1/slope^)-1, where the slope was derived from the plot of the CT value versus the log of the serially diluted template concentration. Maize actin was used as a reference to normalize the amount of transcript. The expression levels of the target genes relative to actin were determined as 2^−ΔCT^ (ΔCT = CT_target_ − CT_actin_).

### Analysis of enzyme activity

A double-antibody sandwich ELISA was used to assay the enzyme levels in the samples. A 100-mg mass of shoots were homogenized in 900 μL of phosphate buffer solution (pH 7.4) in an ice bath and then centrifuged at 4000 rpm for 10 min at 4 °C. The supernatant was transferred to a clean tube, and analysed by plant ent-kaurene oxidase, GA 3-oxidase and GA 2-oxidase (TSZ, USA) following the manufacturer’s instructions. Sample ODs were measured at 450 nm with an Infinite M200 Pro (TECAN, Switzerland), and concentrations were calculated by comparison to sample ODs in the standard curve.

### Statistical analysis

Statistical analysis was conducted using the SPSS statistical software package version 16.0 (IBM Corp, Armonk, NY, USA). First, the effects of the fungicide on physiological and biochemical parameters and the relative expression levels of genes were analysed by one-way analysis of variance (ANOVA) followed by Duncan’s multiple range test. Then, the main and interactive effects of the dose and formulation were analysed using multivariate analysis of variance (MANOVA). Each assay was repeated at least three times. *P* < 0.05 was considered statistically significant in all assays.

## Additional Information

**How to cite this article**: Yang, L. *et al.* The role of gibberellins in improving the resistance of tebuconazole-coated maize seeds to chilling stress by microencapsulation. *Sci. Rep.* **6**, 35447; doi: 10.1038/srep35447 (2016).

**Publisher’s note**: Springer Nature remains neutral with regard to jurisdictional claims in published maps and institutional affiliations.

## Supplementary Material

Supplementary Information

## Figures and Tables

**Table 1:** Germination rates of maize seeds coated with different doses of FS and CF under chilling stress.

Dose (g AI kg^−1^ seed)	Germination rate of FS	Germination rate of CF
0	97.8 ± 2.2^a^	97.8 ± 2.2^a^
0.06	97.8 ± 2.2^a^	100 ± 0^a^
0.12	95.6 ± 2.2^a^	97.8 ± 2.2^a^
0.24	91.1 ± 2.2^a^	97.8 ± 2.2^a^
0.4	88.9 ± 1.2^ab^	97.8 ± 2.2^a^
0.6	64.4 ± 11.1^b^	95.6 ± 3.4^a^

The germination rate was determined 8.5 d after sowing. The seeds were coated with tebuconazole (FS or CF) at 25 °C/20 °C (12 h of light, 12 h of dark) for 60 h, followed by 6 °C/17 °C (12 h of dark, 12 h of light) for 6 d. At least 50 seeds were cultured for each treatment. AI = active ingredient, FS = flowable concentrate for seed treatment, CF = capsule suspension for seed treatment. The results were presented as the means ± SE. The control treatment was set at zero. Different letters indicated significant differences at P < 0.05 (one-way ANOVA, Duncan’s multiple range test).

**Table 2:** MANOVA of the effects of dose, formulation, and their interaction (Dose × Formulation) on the properties of maize seeds and shoots.

Dependent Variable	Dose	Formulation	Dose × Formulation
Germination rate	*	*	*
Shoot length	*	*	*
Fresh weight	*	*	*
GA_3_	*	*	*
GA_4_	*	*	*
GA_1_	*	*	*
KO activity	*	*	*
GA3ox activity	*	*	*
GA2ox activity	NS	*	*
*ZmKO1*	*	*	*
*ZmKO2*	*	*	*
*ZmGA3ox1*	*	*	*
*ZmGA3ox2*	*	*	*
*ZmGA2ox1*	*	*	*
*ZmGA2ox4*	*	*	*
*ZmGA2ox5*	*	*	NS
*ZmGA2ox6*	*	*	*
*ZmGA2ox7*	*	*	*
*ZmGA2ox8*	*	*	*
*ZmGA2ox9*	NS	*	NS
*ZmGA2ox10*	*	*	NS

MANOVA: Multivariate analysis of variance; italicized letters: the relative expression levels of metabolic genes. Dose (0, 0.06, 0.12, 0.24, 0.4, and 0.6 g AI kg^−1^ seed), formulations (FS = flowable concentrate for seed treatment, CF = capsule suspension for seed treatment), Asterisk (*): significant difference at *P* < 0.05, NS: no significant difference at *P* > 0.05.
