# Supplementary material for: The role of gibberellins in improving the resistance of tebuconazole-coated maize seeds to chilling stress by microencapsulation
Source: Sci Rep. 2016 Nov 7;6:35447. doi: 10.1038/srep35447 (PMC5098164; doi:10.1038/srep35447)
Supplement: Supplementary Information [file srep35447-s1.pdf]

# The role of gibberellins in improving the resistance of tebuconazole-coated maize seeds to chilling stress by microencapsulation

Lijuan Yang, Daibin Yang, Xiaojing Yan, Li Cui, Zhenying Wang, Huizhu Yuan*

## Supplementary Information

**Table S1** Primer used for real-time quantitative PCR

| Gene | Name of primers | Sequence (5'-3') | Accession no. |
|---|---|---|---|
| *ZmKO1* | ZmKO1F | CAATCTGTACGGGTGCAACA | BT042205 |
| | ZmKO1R | CCTCTAGGTGCGAGGTACACAT | |
| *ZmKO2* | ZmKO2F | GGAGGAAGGAGGGCCTGTG | EZ104608 |
| | ZmKO2R | GCTCACAGACCAGAGACCAA | |
| *ZmGA3o×1* | ZmGA3o×1F | GCTCACAGACCAGAGACCAA | JX307637.1 |
| | ZmGA3o×1R | TAGCTGCGGAACGGAATTAG | |
| *ZmGA3o×2* | ZmGA3o×2F | TGGGGTGGTGACAAGGAACA | NM_001153053.1 |
| | ZmGA3o×2R | CCTAAAAGAATGGTGAGCGG | |
| *ZmGA2o×1* | ZmGA2o×1F | TTCACCTTCGGGGAGTACAG | NM_001154780 |
| | ZmGA2o×1R | GCATCCACCGTGAAGTTACA | |
| *ZmGA2o×4* | ZmGA2o×4F | GAGAAAGGTGCAGGAAGACG | EC858802 |
| | ZmGA2o×4R | CTGCACTCTCCTCTCCATTG | |
| *ZmGA2o×5* | ZmGA2o×5F | GAACCCATACACCTCTACCGA | AC198080 |
| | ZmGA2o×5R | GGATGACTACCTTACCCGAAA | |
| *ZmGA2o×6* | ZmGA2o×6F | GTTCGGATACGCCAGCAA | AC200263 |
| | ZmGA2o×6R | CAGCACAGCACGGAAACA | |
| *ZmGA2o×7* | ZmGA2o×7F | ACTTCACATGGGGCGACTAC | NM_001154796 |
| | ZmGA2o×7R | GTGTTGGGGCCGGTCTAT | |
| *ZmGA2o×8* | ZmGA2o×8F | TTATGACGACCGACAGGC | AC210731 |
| | ZmGA2o×8R | TACCCAAGTGAAGCGAGA | |
| *ZmGA2o×9* | ZmGA2o×9F | CCGGCGGTAGGATCGATTAC | AC186515 |
| | ZmGA2o×9R | CACGTACAATACGAGCATGGC | |
| *ZmGA2o×10* | ZmGA2o×10F | GTGCCCTACTTCCTCGTTGA | AC194097 |
| | ZmGA2o×10R | TTGTATTACCCGCTCGTTTG | |
| Maize actin | actinF | TCACCATTGGGTCAGAAAGG | NM_001155179 |
| | actinR | GCCAAAATAGAGCCACCGAT | |
